# Corrigendum: A Frequency-Domain Machine Learning Method for Dual-Calibrated fMRI Mapping of Oxygen Extraction Fraction (OEF) and Cerebral Metabolic Rate of Oxygen Consumption (CMRO_2_)

**DOI:** 10.3389/frai.2021.614245

**Published:** 2021-07-12

**Authors:** Michael Germuska, Hannah Louise Chandler, Thomas Okell, Fabrizio Fasano, Valentina Tomassini, Kevin Murphy, Richard G. Wise

**Affiliations:** ^1^ Cardiff University Brain Research Imaging Centre (CUBRIC), Department of Psychology, Cardiff University, Cardiff, United Kingdom; ^2^ FMRIB, Nuffield Department of Clinical Neurosciences, Wellcome Centre for Integrative Neuroimaging, University of Oxford, Oxford, United Kingdom; ^3^ Siemens Healthcare Ltd., Camberley, United Kingdom; ^4^ Division of Psychological Medicine and Clinical Neurosciences, Cardiff University School of Medicine, Cardiff, United Kingdom; ^5^ Department of Neuroscience, Imaging and Clinical Sciences, “G. D’Annunzio University” of Chieti-Pescara, Chieti, Italy; ^6^ Institute for Advanced Biomedical Technologies, “G. D’Annunzio University” of Chieti-Pescara, Chieti, Italy

**Keywords:** calibrated-fMRI, oxygen extraction fraction, CMRO_2_, OEF, machine learning

In the original article, there was a mistake in Table 3 as published. The labels for [Hb] and OEF_0_ were swapped. The corrected Table 3 appears below.

**TABLE 3:** Results of a bivariate regression of OEF_0_ against CBF_0_ and [Hb] for 30 healthy volunteers analyzed with the ML (ensemble of MLPs) and rNLS fitting methods.

Predictor	ML β1 (p value)	rNLS β1 (p value)
[Hb]	−1.42 (0.001)	−2.23 (0.001)
CBF	−0.07 (0.44)	−0.37 (0.005)
Intercept	61.95 (<0.001)	89.48 (<0.001)

In the original article, there was an error. Reference to Table 3 in the text had the labels for [Hb] and OEF_0_ swapped. A correction has been made to **Results**, ***In-vivo***, paragraph 5:

“Table 3 reports the results of a bivariate regression of OEF against [Hb] and CBF for both analysis methods. The slopes of the relationship between OEF and [Hb] are similar to that reported in healthy subjects by Ibaraki et al. (2010), −1.75 Hb (g/dL). As per Ibaraki et al. the relationship between CBF and OEF did not reach significance (*p* = 0.44) for the ML approach, however a significant negative correlation was observed in the rNLS analysis (*p* = 0.005). A univariate analysis of CMRO2,0 against CBF0 is consistent with that observed in healthy controls by Powers et al. (2011) (β1 = 0.2) for both analysis methods, β1 = 0.32 (*p* < 0.001) and β1 = 0.24 (*p* < 0.001) for the ML and rNLS approaches respectively.”

The authors apologize for these errors and state that this does not change the scientific conclusions of the article in any way. The original article has been updated.

