# Supplementary figures and images for: A Frequency-Domain Machine Learning Method for Dual-Calibrated fMRI Mapping of Oxygen Extraction Fraction (OEF) and Cerebral Metabolic Rate of Oxygen Consumption (CMRO2)
Source: Front Artif Intell. 2020 Mar 31;3:12. doi: 10.3389/frai.2020.00012 (PMC7116003; doi:10.3389/frai.2020.00012)

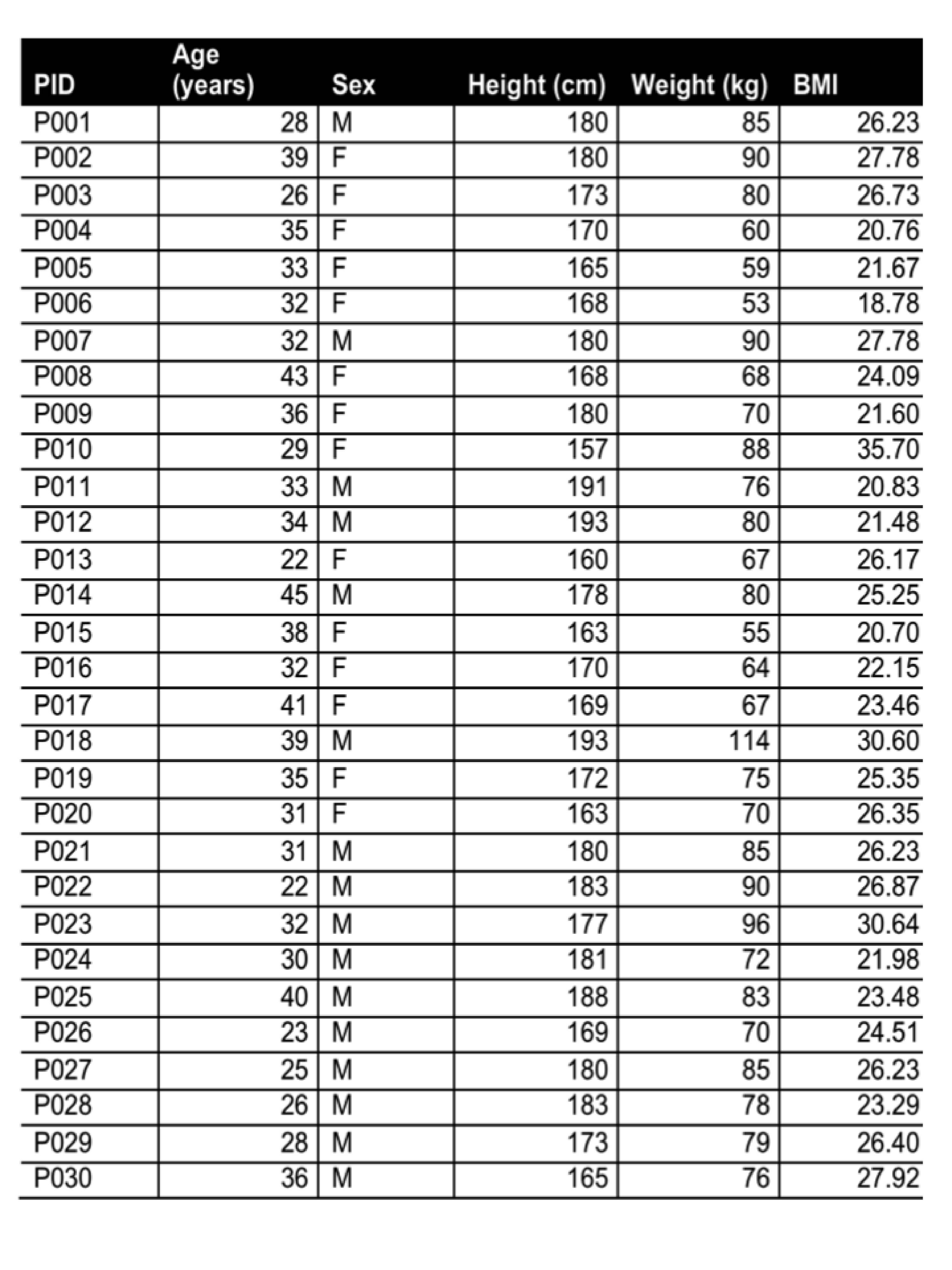

Supplement: Supplementary Table 1 — Demographic data for healthy volunteers. [file Image_1.JPEG]
